# Cat-transmitted Sporotrichosis: A Clonal Household Outbreak with Atypical Human and Canine Disease Due to *Sporothrix brasiliensis*

**DOI:** 10.1007/s11046-026-01075-4

**Published:** 2026-05-12

**Authors:** Bruno Balduino Berber-Freitas, Lara Rocha Batista, Bruno Siqueira Prudente, Leonardo Eurípedes Andrade e Silva, Simone Regina Argenton-Perrella, Érica Rivera Coimbra, Ana Beatriz Oliveira de Souza, Larissa Sousa Araújo, Marilia Prior Fuga, Ivonete Helena Rocha, Jamile Ambrósio de Carvalho, Anderson Messias Rodrigues, Mario León Silva Vergara

**Affiliations:** 1 Tropical Medicine Post Graduate Course, Federal University of Triangulo Mineiro, Uberaba, Minas Gerais Brazil; 2 Infectious Diseases Unit, Internal Medicine Department, Federal University of Triangulo Mineiro, Uberaba, Minas Gerais Brazil; 3 Municipal Zoonosis Control Center, Uberaba, Minas Gerais Brazil; 4 Health Sciences Post Graduate Course, Federal University of Triangulo Mineiro, Uberaba, Minas Gerais Brazil; 5 Laboratory of Emerging Fungal Pathogens, Department of Microbiology, Immunology, and Parasitology, Discipline of Cellular Biology, Federal University of São Paulo (UNIFESP), São Paulo, 04023062 Brazil; 6 National Institute of Science and Technology in Human Pathogenic Fungi, São Paulo, Brazil

**Keywords:** *Sporothrix brasiliensis*, Molecular epidemiology, Household outbreak, Clinical pleomorphism, Cat-transmitted sporotrichosis, One health

## Abstract

The epidemiology of sporotrichosis, a neglected mycosis caused by *Sporothrix* species, has shifted in South America from classic sapronosis to urban zoonosis driven by feline transmission of the hypervirulent *Sporothrix brasiliensis*. However, the fine-scale transmission dynamics of this epidemic, particularly within households, remain poorly understood. Here, we dissect an intrafamilial outbreak in Brazil involving a cat (the index case), a dog, and three humans. High-resolution genotyping using 15 microsatellite markers revealed a clonal transmission event, with isolates from the index cat and human patients being genetically indistinguishable (100% similarity). Multiple bioinformatic analyses, including principal component analysis, minimum spanning tree, and self-organizing maps, corroborated these findings. Strikingly, this outbreak strain produced markedly divergent clinical phenotypes, including Parinaud’s oculoglandular syndrome in an 8-year-old girl, a fixed cutaneous lesion in her 5-year-old brother, and a disseminated maculopapular exanthem in her 28-year-old mother. This clinical pleomorphism from a genetically invariant pathogen unequivocally demonstrates the pivotal role of host factors, such as age, immune response, and inoculation site, in dictating disease manifestation. Furthermore, all outbreak isolates belonged to the *MAT1-1* idiomorph, a finding that challenges the paradigm of a monolithic epidemic dominated by *MAT1-2* strains and suggests a more complex, polycentric expansion of *S. brasiliensis*. Overall, this study provides strong evidence of zoonotic transmission and highlights how host determinants, not pathogen variability, shape the clinical diversity of sporotrichosis.

## Introduction

Sporotrichosis is a neglected subcutaneous mycosis that affects humans and animals and is caused by thermally dimorphic *Sporothrix* species [1, 2]. Historically, this disease has been associated with the classical sapronotic transmission route, where infection results from the traumatic inoculation of fungal propagules from contaminated soil and plant matter [3, 4]. This route has traditionally defined sporotrichosis as an occupational mycosis affecting gardeners, farmers, and forestry workers [5].

Over recent decades, however, an epidemiological paradigm shift has occurred in South America, where sporotrichosis has emerged as a hyperendemic zoonosis driven by cat-to-human transmission [6, 7]. This epidemic, driven by the highly virulent *Sporothrix brasiliensis*, is characterized by rapid geographic expansion and urban hyperendemicity. Central to this process is the domestic cat (*Felis catus*), which functions both as a biological amplifier and as a sentinel host [8]. Infected cats often develop disseminated cutaneous lesions with high fungal loads, transforming them into highly efficient superspreaders capable of transmitting infection to other cats, dogs, and, critically, humans through scratches, bites, droplets and contact with exudates [9–11].

Cat-transmitted sporotrichosis (CTS) now constitutes a distinct epidemiological and clinical entity. Compared with its classical sapronotic form, CTS is more frequently associated with severe, atypical, and refractory disease, presenting a unique one-health challenge. Although Brazil currently represents the epicenter of CTS dissemination [6, 12], familial outbreaks have rarely been described [13]. In this setting, the household becomes the primary locus of transmission, sustained by the convergence of factors such as a highly infectious reservoir host, close and prolonged contact with humans and other animals, and a pathogen of exceptional virulence. Studying intrafamilial outbreaks is therefore crucial for identifying risk factors, determining transmission probabilities, and developing effective prevention strategies. Such investigations require high-resolution molecular tools capable of identifying sources, mapping transmission chains, and distinguishing clonal strains [14, 15].

To address this gap, we applied molecular epidemiology to a well-documented intrafamilial outbreak in Uberaba, Minas Gerais, a region previously considered to have a low incidence of CTS [16]. Using a validated panel of 15 hypervariable microsatellite markers for high-resolution molecular fingerprinting [17], we established the clonal identity of *Sporothrix* isolates and reconstructed transmission pathways linking the feline index case to subsequent human and canine infections. Notably, this outbreak strain produced divergent clinical phenotypes across affected family members. This rare demonstration of clinical pleomorphism from a genetically uniform pathogen highlights that disease expression in sporotrichosis is not solely dictated by pathogen genetics but also by the interplay of host-specific factors (immune status, age, and genetic background) and inoculation dynamics (site, depth, and inoculum burden).

## Methodology

### Study Design and Case Ascertainment

This study describes an intrafamilial CTS outbreak that occurred in 2025 in Uberaba, Minas Gerais, Brazil (19°44′52″ S, 47°55′55″ W). The investigation was initiated following the diagnosis of the first human case at the University Hospital of the Federal University of Triangulo Mineiro. A subsequent epidemiological investigation of the patient’s household identified two additional human cases, one symptomatic dog and one symptomatic cat, the latter being the suspected index case for the outbreak.

### Sample Collection and Fungal Culture

For human cases, clinical samples were collected via sterile swabs from cutaneous or mucosal lesions. For animal cases, samples were obtained through direct imprints of skin lesions. The swabs were placed in Stuart transport medium (CRAL, Brazil) for laboratory processing. The samples were plated in duplicate on Sabouraud dextrose agar (Ionlab, Araucária, PR, Brazil) supplemented with chloramphenicol (Inlab, São Paulo, SP, Brazil) and Mycosel agar (Mbiolog, Contagem, MG, Brazil). The cultures were incubated at both room temperature (25–28 °C) and 37 °C for up to four weeks to observe thermal dimorphism.

### Microscopic and Histopathological Analysis

Cytopathological smears from swabs and lesion imprints were stained via a Romanowsky-type rapid panoptic stain kit (Instant Prov, Newprov, Brazil) [18]. The slides were examined under light microscopy for yeast-like cells consistent with *Sporothrix* spp. [18]. For the deceased canine case, tissue samples were collected postmortem, fixed in formalin, and processed for histopathological analysis to identify fungal elements within the tissue [19].

### Molecular Typing and Bioinformatic Analysis

Genomic DNA was extracted from pure fungal cultures via the FastDNA Kit (MP Biomedicals, USA) [6]. Species-level identification was performed via a triplex-probe quantitative PCR (qPCR) assay targeting the β-tubulin gene, which enables simultaneous detection and differentiation of *S. brasiliensis* (Sbra), *S. schenckii* (Ssch), and *S. globosa* (Sglo), as described by Della Terra et al*.* [20]. The mating-type locus of each isolate was subsequently determined via a duplex PCR assay with specific primers designed to amplify a 673 bp fragment for the *MAT1-1* idiomorph or a 291 bp fragment for the *MAT1-2* idiomorph as described by de Carvalho et al*.* [21].

High-resolution genotypic analysis was then performed via simple sequence repeat (SSR) fingerprinting, following the comprehensive protocol developed by Losada et al*.* [17]. This protocol utilizes a validated panel of 15 hypervariable SSR markers amplified across five multiplex PCRs (M1–M5). The forward primers were labeled with fluorescent dyes (FAM, VIC, or NED), and fragment analysis was conducted via capillary electrophoresis on a SeqStudio Genetic Analyzer (Applied Biosystems, USA) with a LIZ600 internal size standard to ensure precise allele sizing.

Allele calling and all subsequent bioinformatic analyses were conducted via BioNumerics software (version 7.6, Applied Maths, Belgium) [17, 22]. The SSR profiles of the outbreak isolates were integrated into a comparative dataset containing reference strains previously characterized by Losada et al*.* [17], allowing robust phylogenetic contextualization. Genetic relationships were assessed through a multifaceted approach. First, a dendrogram was constructed via the categorical (values) coefficient and the unweighted pair group method with arithmetic mean (UPGMA) clustering algorithm to visualize overall genetic similarity [23].

To further explore these relationships in a multidimensional space and statistically validate the genetic structure, multivariate analysis of variance (MANOVA) [24] and principal component analysis (PCA) [25] were performed. A minimum spanning tree (MST) was generated to infer clonal relationships and visualize the most parsimonious connections between genotypes via the Prim algorithm [26], which is critical for elucidating potential transmission pathways in an outbreak setting. Finally, an unsupervised neural network method, self-organizing maps (SOMs) [27], was applied to explore population structure and identify genetic clusters without preexisting taxonomic assumptions [22].

## Results

**Fig. 1:**
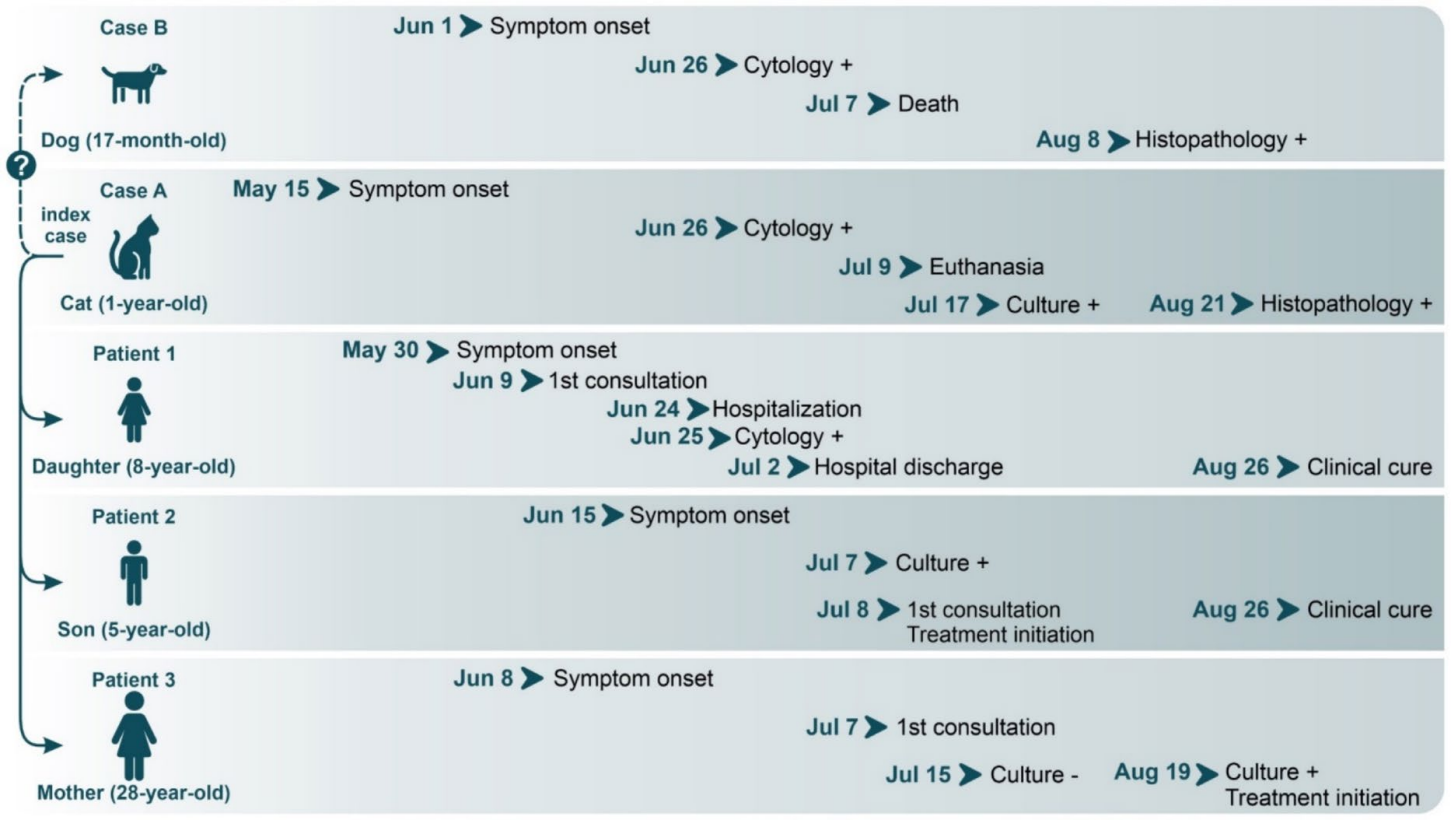
Timeline of the household outbreak of feline-transmitted sporotrichosis, Uberaba, Minas Gerais, Brazil

The household outbreak included three human cases (two children and their mother), one canine case, and one feline case, the latter being the suspected index source. The clinical progression, diagnostic findings, and outcomes for each patient are detailed below and depicted in Fig. 1.

### Clinical Case Descriptions

#### Human‒Animal Interactions and Exposure History

Detailed anamnesis revealed that all three patients maintained close daily contact with the index cat (case A) within the household environment. The family reported handling the animal and treating its cutaneous wounds without personal protective equipment prior to the diagnosis. Despite this high-risk exposure to fungal exudates, none of the patients recalled specific traumatic events, such as bites or scratches. This pattern of interaction supports the hypothesis of transmission via direct contact with secretions or contaminated fomites, which is characteristic of the close coexistence described in the One Health framework.

#### Patient 1: 8-year-old Female

**Fig. 2:**
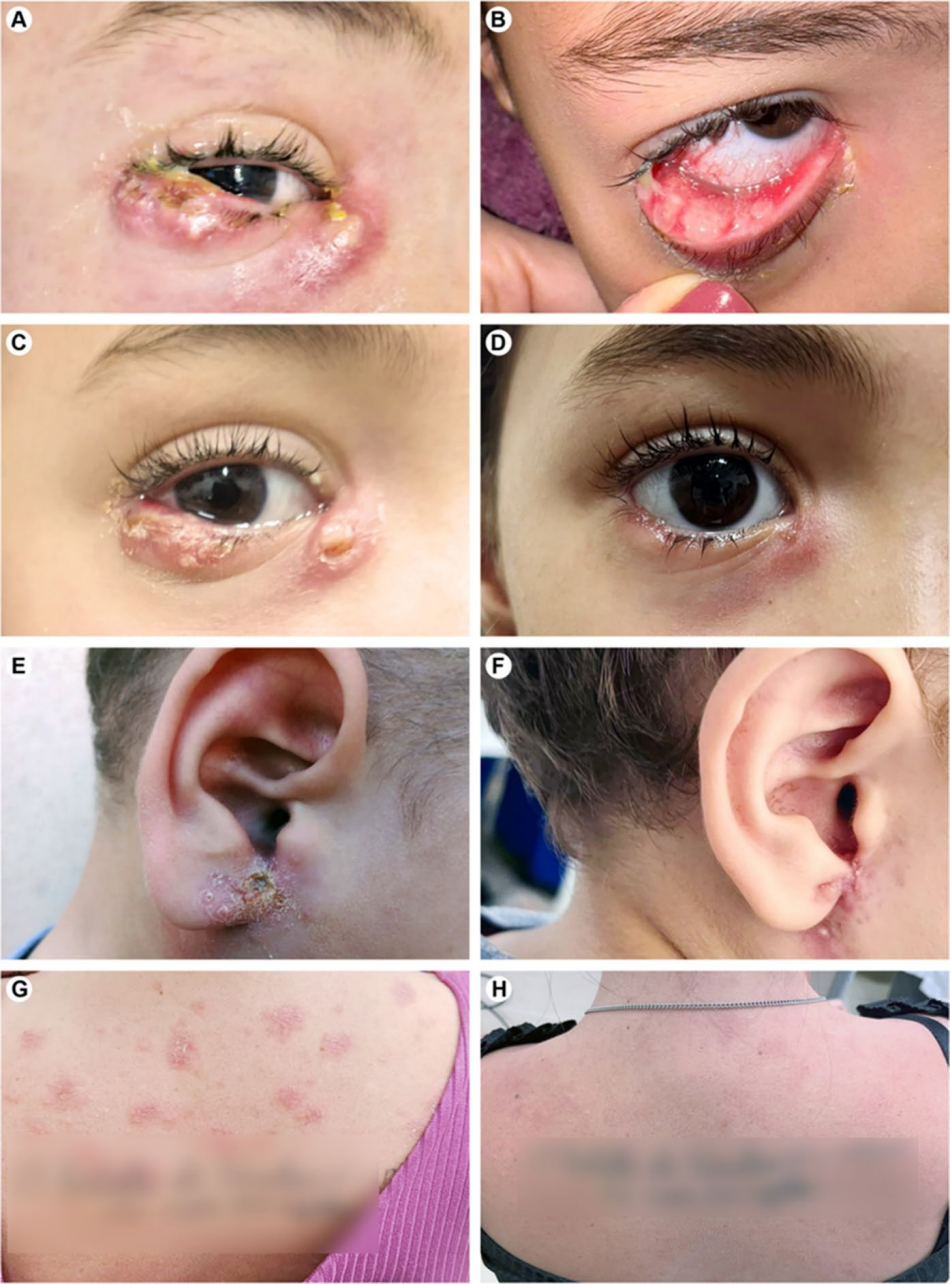
Clinical presentation and evolution of sporotrichosis cases. **A**, **B** Patient 1 at admission, showing right lower blepharitis, dacryocystitis, and purulent discharge, with microabscess formation on the lower tarsal conjunctiva. **C**, **D** Follow-up of the ocular lesion at 15 and 52 days, respectively, after initiating itraconazole therapy (200 mg/day). **E** Patient 2 presented with an inflammatory right infraauricular lesion with ulceration, purulent discharge, and perilesional scaling. **F** Resolution of the lesion in Patient 2 after four weeks of itraconazole treatment (200 mg/day). **G** Patient 3, initial presentation of cutaneous lesions, characterized by erythematous, scaling, and pruritic maculopapular rash. **H** Improvement in the cutaneous lesions of Patient 3 after 15 days of itraconazole therapy (200 mg/day)

An 8-year-old female presented to the ophthalmology emergency service with a 10-day history of a painful, progressive nodule on her right lower eyelid, which had developed a purulent discharge over the preceding seven days. Physical examination revealed a nodule exhibiting classic signs of inflammation (calor, rubor, and localized pain on palpation), in addition to purulent discharge, dacryocystitis, and microabscesses on the inferior tarsal conjunctiva (Figs. 2A and B). An initial diagnosis of preseptal cellulitis was made, and the patient was prescribed oral amoxicillin-clavulanate and ibuprofen.

Despite 14 days of antibiotic therapy and close outpatient monitoring, her condition deteriorated, as indicated by worsening edema, increased discharge, and the emergence of painful ipsilateral preauricular lymphadenopathy. This clinical failure prompted hospitalization for further investigation and management. A cranial computed tomography (CT) scan confirmed right preseptal cellulitis, detailing diffuse thickening of the eyelid and revealing a 1 × 1 cm septated collection adjacent to the nasolacrimal duct ostium. Concurrently, a cervical ultrasound identified multiple reactive lymph nodes. The antibiotic regimen was escalated to intravenous vancomycin and meropenem.

The failure to respond to this antibacterial regimen prompted a diagnostic re-evaluation, which was informed by a critical epidemiological link provided by the patient’s mother during hospitalization. She reported that the family had a cat with extensive skin lesions and that another child had an ulcerated lesion on his ear. This information, coupled with the refractory nature of the infection, shifted the diagnostic focus. Ocular sporotrichosis, presenting as Parinaud’s oculoglandular syndrome, was suspected. Swabs from ocular secretions were collected for direct microscopy and fungal culture. Both tests were positive for *Sporothrix* spp., confirming the diagnosis. The corresponding isolate was deposited in our collection under the code Ss1251. Treatment was immediately initiated with oral itraconazole (200 mg/day). The patient demonstrated substantial clinical improvement within one week (Figs. 2C and D), allowing her to be discharged for continued outpatient management. At present, the lesions have remitted, and she remains on antifungal therapy under regular follow-up.

#### Patient 2: 5-year-old Male

The 5-year-old brother of Patient 1 presented with a three-week history of a progressive auricular lesion, initially appearing as papules, then evolving into pustules, and subsequently forming a crust. Physical examination revealed an inflammatory, ulcerative infraauricular lesion with perilesional edema and scaling, accompanied by nontender, unilateral cervical lymphadenopathy (Fig. 2E). Prompted by his sister’s diagnosis one week prior, a fungal culture had already been collected from the lesion, which returned positive for *Sporothrix* spp., and the corresponding isolate was deposited in our collection under the code Ss1250. He was started on oral itraconazole (200 mg/day). After four weeks of therapy, the lesion showed significant signs of cicatrization and healing (Fig. 2F).

#### Patient 3: 28-year-old Female

The 28-year-old mother of the two children (Patients 1 and 2) presented with a four-week history of erythematous, scaling, and intensely pruritic maculopapular exanthem distributed across her back, chest, and upper limbs (Fig. 2G). She had previously used antihistamines with only minimal symptomatic relief. Following her children’s confirmed diagnoses, initial fungal swabs from her skin lesions were collected but yielded negative results for fungi. Over the subsequent two weeks, as the rash persisted and disseminated to her abdomen and lower limbs, a repeat culture was performed. This second sample was positive for *Sporothrix* spp., and the corresponding isolate was deposited in our collection under the code Ss1249. Treatment with oral itraconazole (200 mg/day) was initiated, resulting in marked clinical improvement and complete resolution of pruritus within two weeks (Fig. 2H).

### Animal Cases

**Fig. 3:**
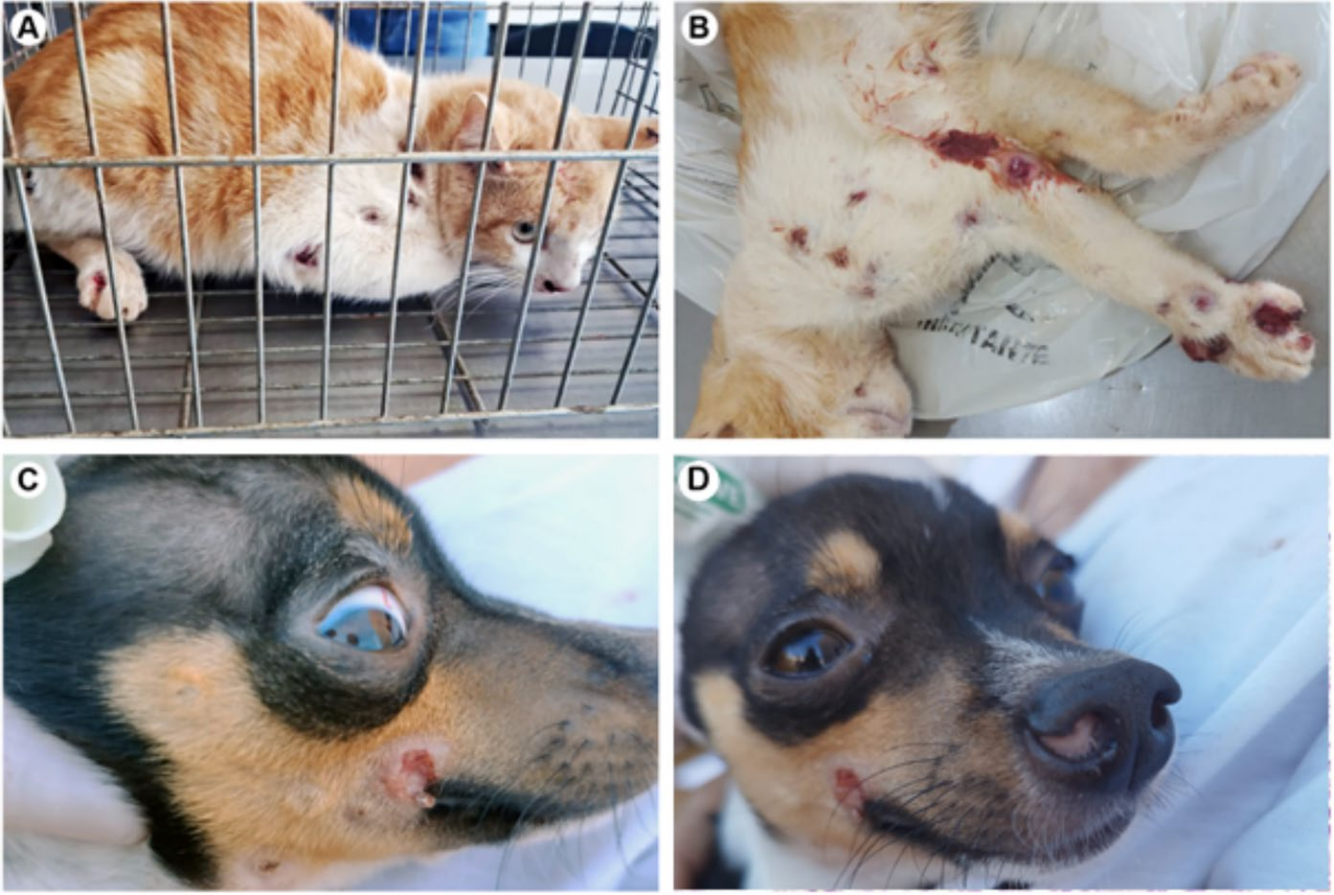
Clinical and histopathological findings in animal cases of sporotrichosis. **A**, **B** Case A, a 1-year-old, male, intact, mixed-breed feline, presented with multiple ulcerated cutaneous lesions on the thoracic and pelvic limbs, head, and nasal planum. **C**, **D** Case B, a 17-month-old male mixed-breed dog, presented with an ulcer on the right labial commissure and right nasal nodule

The family cat (case A), a 1-year-old, male, intact, mixed-breed feline, presented with a 30-day history of progressive wounds appearing all over its body (Figs. 3A and B). The owner reported that the animal had constant outdoor access and was in contact with stray cats. The physical examination revealed multiple ulcerated cutaneous lesions on the thoracic and pelvic limbs, which appeared to follow a lymphatic distribution. Additional ulcers were present on the head, ear, nasal plane, dorsum, and tail. No respiratory symptoms were reported at the initial presentation. A lesion imprint for direct microscopy and a swab for fungal culture were performed. Direct examination revealed structures compatible with *Sporothrix* spp. however the culture was negative.

Twenty days after the initial diagnosis, the owners requested that the animal be euthanized due to severe deterioration of its clinical condition. Before euthanasia, a new sample was collected for culture and processing. The subsequent necropsy revealed yeast-like structures in the skin, axillary lymph nodes, and lungs, characterizing disseminated sporotrichosis. The second fungal culture ultimately yielded a positive result for *Sporothrix* spp. (isolate Ss1248).

The family dog (case B), a 17-month-old, male, intact, mixed-breed canine, cohabited with the index cat and had a history of close physical contact with the feline. The dog presented with a 20-day history of a lesion on its mouth and a nodule on its nose. Physical examination revealed a nodule in the right nasal vestibule, an ulcer on the right labial commissure, and a crusted lesion in the ipsilateral submandibular region. A swab was collected for direct microscopy and fungal culture. Direct microscopic examination revealed positive results for yeast-like structures, but the initial fungal culture was negative. The animal died from unrelated trauma two weeks later. Postmortem histopathology of the skin lesions confirmed ulcerative dermatitis with multifocal lymphohistiocytosis and the presence of fungal elements morphologically compatible with *Sporothrix* spp. (Fig. 3D).

### Molecular Characterization of the Outbreak

All fungal isolates recovered from the three human patients (Ss1249, Ss1250, and Ss1251) and the index cat (Ss1248) were subjected to molecular analysis to confirm species identity and determine their genetic relatedness. A triplex-probe quantitative PCR (qPCR) assay confirmed that all the isolates were *S. brasiliensis*, with positive amplification resulting exclusively from the Sbra probe (Cqs 30.34, 30.45, 36.59, 37.20). Subsequent mating-type determination via duplex PCR revealed that all the isolates uniformly harbored the *MAT1-1* locus (673 bp fragment).

**Fig. 4:**
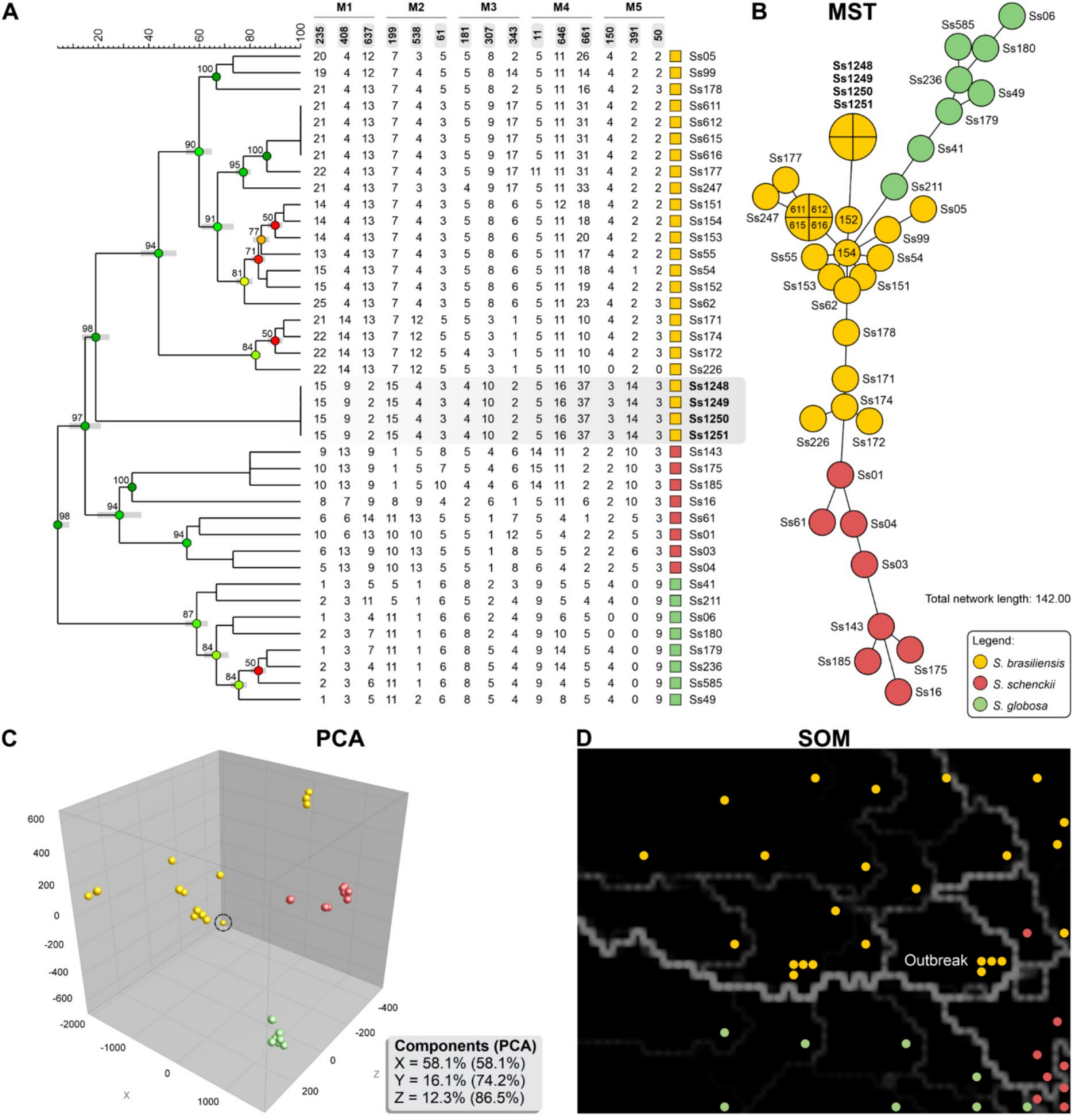
Molecular genotyping of *Sporothrix brasiliensis* isolates from the Uberaba outbreak. **A** UPGMA dendrogram based on 15 SSR markers, showing that all four outbreak isolates (index cat and three human cases, highlighted in gray) are genetically indistinguishable with 100% similarity (Ss1248, Ss1249, Ss1250, and Ss1251). **B** A minimum spanning tree (MST) collapses the outbreak isolates into a single node, indicative of a clonal complex. **C** 3D principal component analysis (PCA) showing the colocalization of the four outbreak isolates (circle) within the broader *S. brasiliensis* population cluster (yellow spheres). **D** A self-organizing map (SOM) projects all four outbreak isolates onto a single cell (a hexagon named ‘outbreak’), confirming their identical genotype

To evaluate the genetic relatedness of the Uberaba outbreak isolates and situate them within a broader national context, we performed high-resolution genotyping using 15 SSR markers. For this analysis, the SSR profiles from the outbreak isolates were integrated into a comparative dataset containing reference strains previously characterized by Losada et al*.* [17]. The resulting UPGMA dendrogram revealed that the isolates from the index cat and all three human patients were molecularly indistinguishable, forming a single monophyletic clade with a global cophenetic value of 98 and a similarity coefficient of 100% (Fig. 4A).

Multiple bioinformatics approaches corroborated this genetic homogeneity. In agreement with the dendrogram, the minimum spanning tree (MST) analysis collapsed all four isolates into a single node (total network length = 142.00; Fig. 4B). In the PCA, the genetic structure of the *Sporothrix* clinical clade was clearly resolved, and PC1 effectively segregated the *S. brasiliensis* population from the *S. schenckii* and *S. globosa* clusters. In contrast, the second component (PC2) further differentiated *S. globosa* from *S. schenckii*. Within this established population structure, all outbreak isolates colocalized deep inside the *S. brasiliensis* cluster. This genetic homogeneity is depicted in the overall 3D-PCA results, where the first three principal components explained 58.1%, 16.1%, and 12.3% of the total variance, respectively (Fig. 4C). The clear species-level segregation observed in the PCA validates the initial qPCR identification and is founded on the high discriminatory power of the SSR panel. This resolution was confirmed by a MANOVA performed on the comprehensive comparative dataset, which revealed highly significant genetic differentiation among the three major clinical *Sporothrix* species (Wilks’ lambda, F(30, 46) = 65.902, *p* < 0.001). This statistical validation of the markers in the reference population confirms the robustness of the distinct clustering observed for the outbreak isolates. Univariate ANOVAs revealed that 13 of the 15 individual SSR markers were significant contributors to this difference (*p* < 0.05).

Finally, to further explore the genetic topology in an unsupervised manner, a self-organizing map (SOM) was generated (Fig. 4D). Neural networks project them onto the same cell of the Kohonen map, confirming that they belong to the same genotype. The cell containing this clonal complex was separated from closely related genotypes, including isolates from Minas Gerais (Ss05) and Rio de Janeiro (Ss177), by faint, dark lines, indicating minor genetic divergence but a shared recent ancestry. In contrast, thick, bright white lines demarcated the group from more distantly related lineages, highlighting its distinct position within the broader *S. brasiliensis* population structure. Collectively, these results provide strong molecular evidence for the zoonotic transmission of a single *S. brasiliensis* strain from the index cat to human family members. The etiologic agent could not be isolated from the canine case for genotyping.

## Discussion

Our study offers a detailed clinical and molecular characterization of an intrafamilial outbreak of cat-transmitted sporotrichosis, providing critical insights into the fine-scale transmission dynamics of *S. brasiliensis*. These findings are situated within the broader epidemiological shift in South America, where sporotrichosis has transitioned from classic sapronotic mycosis to hyperendemic urban zoonosis [7, 10, 12, 17, 18].

Driven by highly virulent *S. brasiliensis* and facilitated by cat transmission, this epidemic now represents a significant public health challenge, with Brazil as its epicenter [10, 28]. Despite the scale of this phenomenon, transmission pathways and genetic relationships within discrete outbreak settings remain poorly defined [29, 30]. The present report from Uberaba, a municipality in Minas Gerais with a historically low number of CTS notifications, underscores both the geographic expansion of the disease and its frequent underreporting, highlighting the urgent need for enhanced surveillance.

The basis of this investigation is the molecular confirmation of a clonal outbreak, advancing beyond epidemiological associations to provide strong evidence of a single-source transmission event. High-resolution genotyping with 15 hypervariable SSR markers revealed that all the isolates from the index cat and three human patients were indistinguishable, yielding a 100% similarity coefficient. The strength of this approach lies in the discriminatory capacity of SSR loci [17], which enables differentiation between closely related isolates, a resolution that is critical for outbreak investigations [15, 17, 31].

The results of the multimodal bioinformatic analyses reinforced this conclusion. PCA placed all four outbreak isolates in a tightly colocalized cluster within the broader *S. brasiliensis* population structure, which was distinctly segregated from other medically relevant *Sporothrix* [17, 22]. MST analysis further collapsed the outbreak isolates into a single node, graphically illustrating their clonal relationship [17, 22, 29, 30]. Finally, an unsupervised self-organizing map independently corroborated these results, mapping all the isolates to the same cell without prior clustering assumptions [27, 32, 33].

The clonal nature of this outbreak reflects the broader population dynamics of *S. brasiliensis* [22, 29, 34, 35]. Population genetic studies consistently demonstrate that its zoonotic expansion is driven predominantly by the spread of low-diversity clones, resulting in geographically structured populations of highly related genotypes [14, 18, 36–40]. This founder-effect propagation contrasts with the more recombinant population structure of the sapronotic *S. schenckii* [17, 22, 28, 34, 35]. Thus, time has emerged as a critical factor in shaping the dynamics of *S. brasiliensis* expansion, with the absence of sanitary barriers facilitating the introduction of new genotypes into transmission networks over time [41]. The present study thus provides a microepidemiological illustration of how a single clone can establish within a domestic environment and be efficiently transmitted across family members [29].

A striking finding is the diverse clinical spectrum observed among individuals infected with a single, genetically invariant pathogen. This outbreak highlights the clinical pleomorphism of sporotrichosis, with the *S. brasiliensis* genotype held constant, thereby revealing the decisive role of host-specific factors in shaping disease outcomes. The three human cases exemplify this paradox: an 8-year-old girl developed Parinaud’s oculoglandular syndrome, a 5-year-old brother presented with a fixed cutaneous lesion, and a 28-year-old woman presented with a pruritic maculopapular exanthem. Each presentation highlights distinct host–pathogen interactions.

Ocular sporotrichosis, which is increasingly recognized during the *S. brasiliensis* epidemic, typically results from the direct inoculation of the fungus into the conjunctival mucosa, which triggers a potent granulomatous inflammatory response [42–45]. Pediatric populations appear to be particularly susceptible, with higher reported rates of associated dacryocystitis in children [42, 44, 46]. In contrast, the fixed cutaneous lesion represented a more contained form of the disease. This presentation is thought to occur in hosts that mount a robust and effective cell-mediated immune response, which successfully restricts the pathogen to the initial site of inoculation, thereby preventing lymphatic spread [47].

The disseminated, pruritic maculopapular exanthem is the most atypical clinical presentation in this outbreak. This clinical picture does not align with the classic hematogenous dissemination typically observed in severely immunocompromised individuals, nor does it fit neatly into the described categories of hypersensitivity reactions associated with sporotrichosis, such as erythema nodosum or Sweet’s syndrome [48–50]. The widespread and highly pruritic nature of the rash in this immunocompetent adult was initially suggestive of a hyperergic or id-like reaction, supported by negative fungal cultures at onset. However, the subsequent isolation of *S. brasiliensis* from persistent lesions confirmed that, despite the atypical maculopapular appearance, the pathogen successfully established active cutaneous infection sites, likely following a period of autosensitization [3, 51, 52].

These divergent outcomes, arising from an outbreak strain, provide compelling evidence that host factors, such as age, immune response, inoculation site, inoculum size, and genetic background, predominate in determining clinical manifestations [3, 52–55]. By eliminating pathogen variability as a confounder, this household cluster allows the attribution of clinical diversity almost exclusively to host determinants and transmission routes [56]. Murine models corroborate this observation, demonstrating that the route of inoculation critically influences disease outcome, with intravenous challenge leading to disseminated, frequently lethal infection [53], whereas subcutaneous inoculation produces a localized form with minimal dissemination [55]. Together, these findings challenge pathogen-centric models of virulence and highlight the importance of investigating host genetics and immunopathogenesis, in line with the modern concept of “deep phenotyping” [57]. Notably, such dynamics are not unique to sporotrichosis. Infections caused by clonal or genetically homogeneous pathogens, such as *Mycobacterium tuberculosis* [58], *Leishmania brasiliensis* [59], *Cryptococcus gattii* [60], and *Candidozyma auris* [61], consistently demonstrate that host factors dictate the clinical spectrum, from asymptomatic carriage to severe disease.

In addition to elucidating host–pathogen interactions, this study contributes novel insights into the molecular epidemiology of *S. brasiliensis*. All the isolates carried the *MAT1-1* locus, a noteworthy finding given that the decades-long epidemic wave from Rio de Janeiro is dominated by *MAT1-2* strains [21, 62]. The identification of a transmissible *MAT1-1* clonal complex in Minas Gerais challenges the prevailing model of a single monolithic epidemic and suggests a more complex, polycentric scenario [38, 63]. Possible explanations include (i) an independent founder event of *MAT1-1* within Minas Gerais [38, 63]; (ii) long-range dispersal from regions such as Rio Grande do Sul, where *MAT1-1* is more common [6, 12]; or (iii) rare sexual recombination generating novel clonal lineages [21, 62]. Regardless of origin, these findings highlight the need for systematic, nationwide molecular surveillance to map genetic diversity, population structure, and dispersal routes. Such efforts are critical, as different lineages may differ in virulence or antifungal susceptibility [41].

The index cat exhibited severe disseminated disease with a high fungal burden, effectively functioning as a superspreader [64]. The absence of traumatic inoculation in human cases supports the role of nontraumatic transmission routes, including contact with exudates, contaminated fomites, or possibly aerosols, routes that remain poorly characterized [11, 65, 66]. The concurrent infection of the family dog, confirmed histopathologically, further illustrates the intense transmission pressure in environments contaminated by a severely ill cat host.

This report leverages advanced molecular tools to dissect an intrafamilial outbreak of *S. brasiliensis*, providing unequivocal evidence of clonal interspecies transmission from a cat source. These findings demonstrate the broad clinical spectrum of infection and show that host factors and transmission routes, rather than pathogen variability, are the primary drivers of outcomes. The detection of a *MAT1-1* clonal complex beyond its previously recognized range adds nuance to the epidemiological landscape of *S. brasiliensis*. Collectively, these insights reinforce the urgency of a unified health strategy for the surveillance and control of this escalating public health threat.

## Data Availability

No datasets were generated or analysed during the current study.
